# Perceived ankle instability is not related to ankle joint position sense, movement detection and inversion/eversion peak power: an observational study

**DOI:** 10.1186/1757-1146-5-S1-O52

**Published:** 2012-04-10

**Authors:** Fereshteh Pourkazemi, Claire Hiller, Jacqueline Raymond, Kathryn Refshauge

**Affiliations:** 1 School of Physiotherapy, Faculty of Health Scineces, University of Sydney, NSW, Australia; 2 School of Exercise and Sport Sciences, Faculty of Health Scineces, University of Sydney, NSW, Australia

## Background

It is not known why continuing instability exists after ankle sprain. The most common hypotheses include impairments in proprioception, muscle power or postural control [1] but a relationship has not been established. Therefore, the aim of this study was to investigate the relationship between functional instability and invertor/evertor peak power, accuracy of movement detection and threshold for position sense in the ankle.

## Materials and methods

Sixty three participants with history of either no ankle sprain or only one ankle sprain were recruited. Functional ankle instability was measured using the Cumberland Ankle Instability Tool (CAIT), a highly reliable measure of functional ankle instability [2]. Invertor/evertor power testing was performed using a Biodex isokinetic dynamometer at speeds of 30, 60 and 120°/sec and the scores were normalised using participants’ BMI. Joint position sense was measured by actively matching the 3 test angles in inversion and eversion with the contra lateral ankle. Movement detection sense was tested at three velocities, 0.1, 0.5, and 2.5°/sec, in a random order. The relationship between perceived ankle instability and proprioception or inversion/eversion peak power was investigated using Pearson product-moment correlation coefficient. Preliminary analyses were performed to ensure the assumptions of normality, linearity and homoscedasticity were not violated.

## Results

No correlation was found between the CAIT scores and the three measured variables. The strongest correlation was between CAIT score and inversion peak power at 30°/s (r=0.220, p= 0.083).

## Conclusion

Based on our findings, functional ankle instability (as measured by CAIT) is not related to inversion/eversion peak power, or with joint movement detection or position sense at the ankle. These findings are consistent with the results of previous studies investigating the relationship between CAIT score and other functional tests [3]. The lack of a relationship suggests that impaired proprioception and muscle power do not explain perceived ankle instability.
